# Supplementary material for: Quantitative proteomic landscape of metaplastic breast carcinoma pathological subtypes and their relationship to triple-negative tumors
Source: Nat Commun. 2020 Apr 7;11:1723. doi: 10.1038/s41467-020-15283-z (PMC7138853; doi:10.1038/s41467-020-15283-z)
Supplement: Supplementary file 1 — Supplementary Information [file 41467_2020_15283_MOESM1_ESM.pdf]

Supplementary Information

Djomehri et al. Quantitative proteomic landscape of metaplastic breast carcinoma pathological subtypes and their relationship to triple-negative tumors

Supplementary Figure 1

A

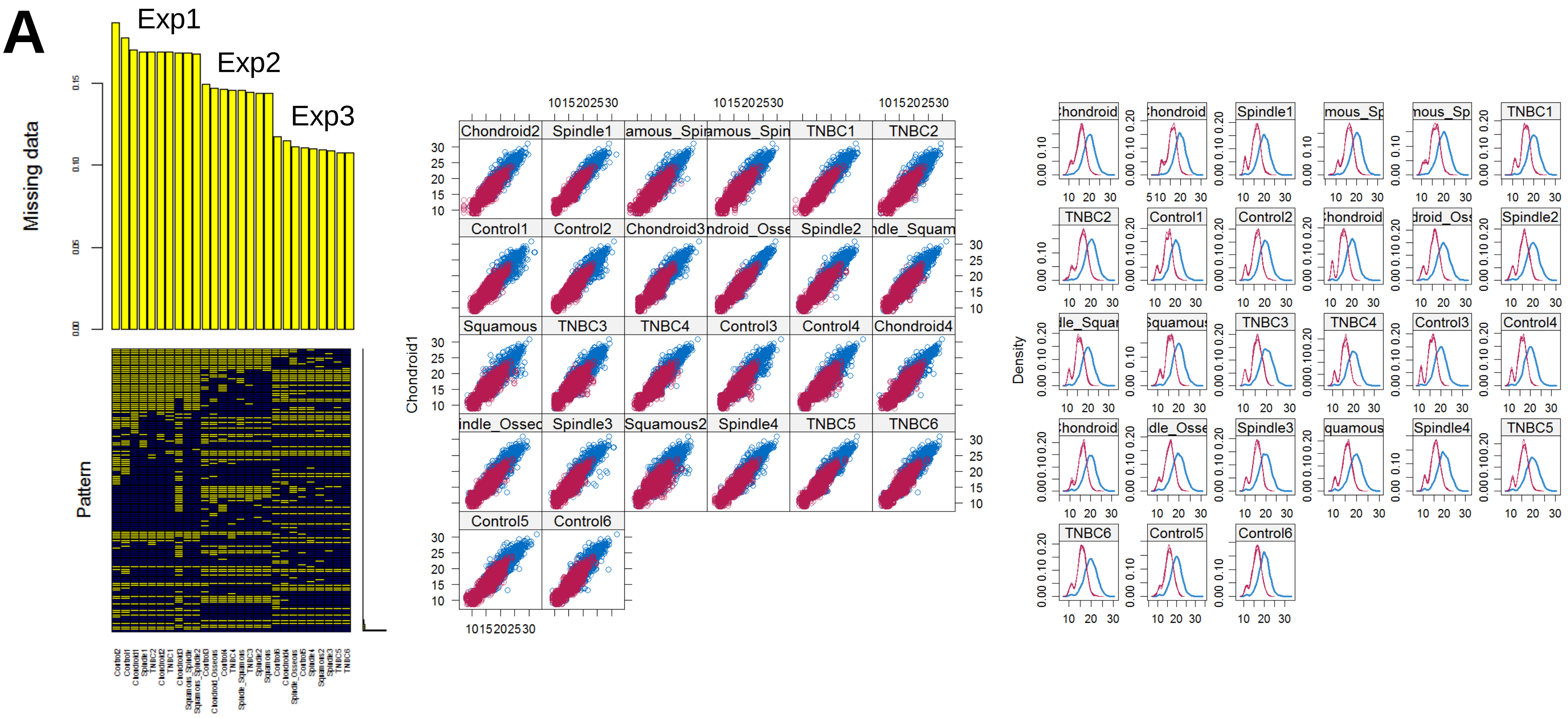

B

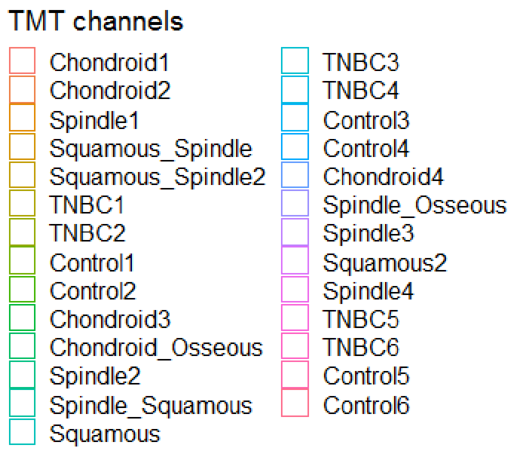

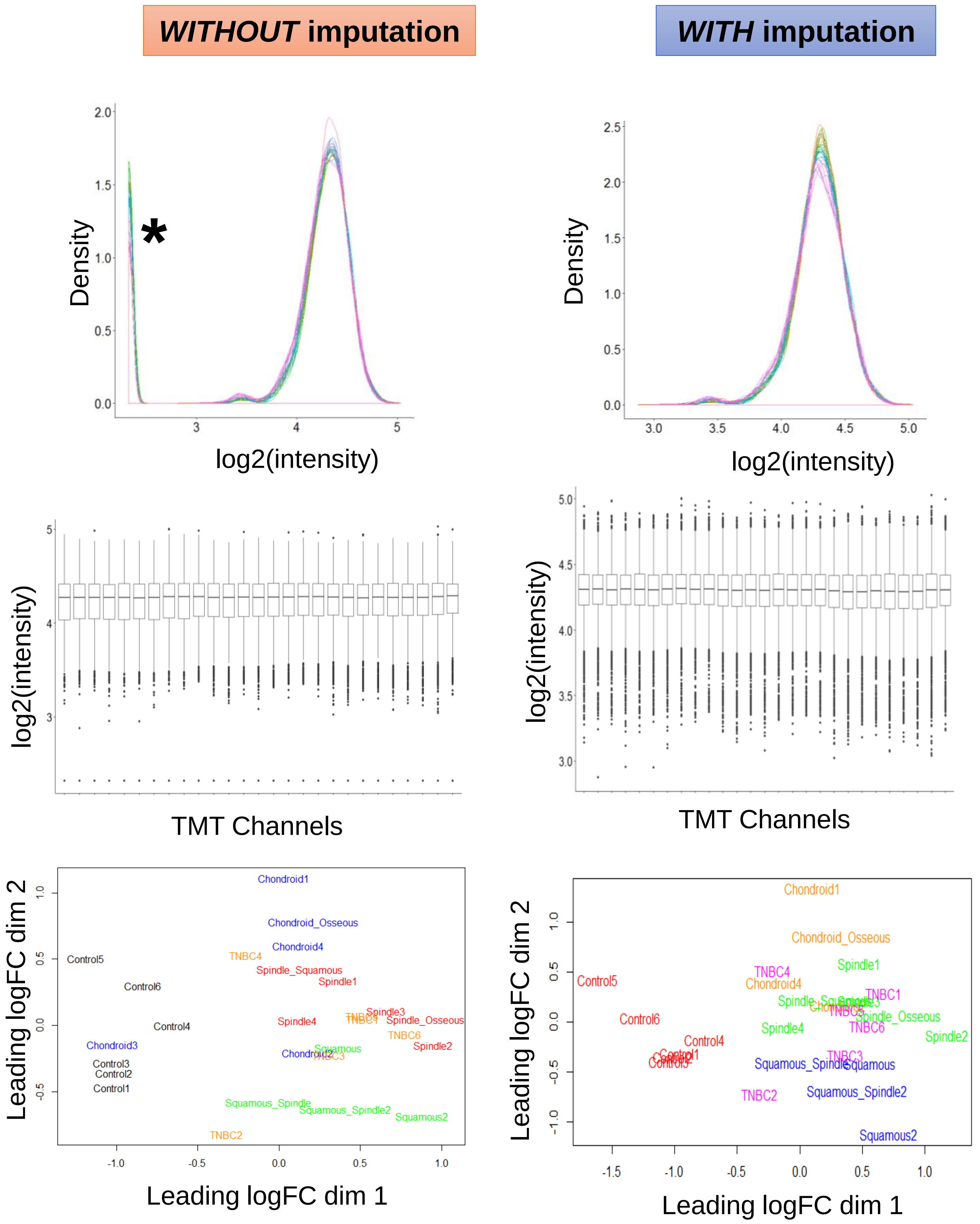

	estimate	std.error	p.value
(Intercept)	-2.4E-01	7.7E-02	3.1E-03
Chondroid2	-4.9E-02	1.9E-02	1.2E-02
Spindle1	2.1E-01	1.9E-02	0.0E+00
Squamous_Spindle	1.3E-01	1.6E-02	2.1E-10
Squamous_Spindle2	6.6E-02	2.0E-02	1.6E-03
TNBC1	2.2E-01	2.2E-02	3.1E-15
TNBC2	-4.3E-02	2.0E-02	3.2E-02
Control1	-4.9E-02	2.5E-02	6.0E-02
Control2	1.5E-01	3.6E-02	2.0E-03
Chondroid3	2.3E-02	4.1E-02	5.8E-01
Chondroid_Osseous	3.1E-01	1.8E-02	0.0E+00
Spindle2	9.1E-02	2.3E-02	1.0E-03
Spindle_Squamous	5.3E-02	2.2E-02	2.7E-02
Squamous	2.8E-02	1.7E-02	1.0E-01
TNBC3	-5.3E-02	2.3E-02	3.8E-02
TNBC4	3.3E-02	2.0E-02	1.0E-01
Control3	-1.4E-01	3.5E-02	1.1E-03
Control4	4.4E-03	2.9E-02	8.8E-01
Chondroid4	1.3E-01	1.6E-02	2.6E-12
Spindle_Osseous	8.3E-02	1.9E-02	9.3E-05
Spindle3	-1.2E-01	1.7E-02	5.1E-12
Squamous2	-2.4E-01	1.9E-02	4.1E-10
Spindle4	1.9E-01	2.3E-02	1.5E-06
TNBC5	-9.3E-02	2.1E-02	4.5E-05
TNBC6	-1.3E-02	2.3E-02	5.8E-01
Control5	1.4E-01	1.9E-02	1.6E-07
Control6	-5.3E-02	2.3E-02	3.3E-02

p < 1.6E-03

Supplementary Figure 1. Pre-processing of LC-MS/MS TMT 10-plex proteomics data before and after data imputation and batch correction.
A. Missing data percentages and patterns in each of the three experiments 1, 2, and 3 (Exp = experiment) contained 18%, 14%, and 12% missing values, respectively. Scatter plots (middle) show one variable against all others (i.e. 26 comparisons between patients) and right plots are densities of imputed data against experimental data. The 'mice' package was used in R (v3.4.0) with the parameters: m=5 (number of imputed data sets), method=pmm (predictive mean matching), and maxit=50 (number of iterations). The multivariate imputation by chained equations method is a gold standard data imputation algorithm that accounts for uncertainty in the data when values are missing at random following Rubin's Rules
B. Comparison of the raw data distribution of 27 patient samples with and without data imputation. Density distribution plots (top and middle) showing log2-transformed protein abundances for all TMT channels of 3 experiments, and an MDS scatter plot (bottom) of computed distances between protein expression profiles of all samples. The asterisk (*) indicates the peak of missing values without data imputation. The table shows statistical performance (pooled estimates, two-sided p-values) of imputed data sets after fitting to a linear model and pooling the data to attain a median p-value<1.6E-03. The MS/MS spectra were first searched using MSFragger (v20181128) database search tool against UniProt human protein database (UP000005640), followed by processing by PeptideProphet with high-mass accuracy binning and semi-parametric mixture modeling to compute correct identification for each peptide-to-spectrum (PSM) searching. PSM lists were created for each TMT 10-plex experiment, and protein groups were assembled in ProteinProphet filtered to 1% false discovery rate (FDR). PSMs passing through filters in the Philosopher label-free quantification module corresponded to TMT reporter ion intensities, and used as input to TMT-Integrator for normalization to the reference channel and log2 transformation.

Supplementary Figure 2

Method	Optimal # Clusters
Bayesian	2
WSS	2
Vegan	2
Elbow	3
Gap Statistic	3
Silhouette	4
Apcluster	4

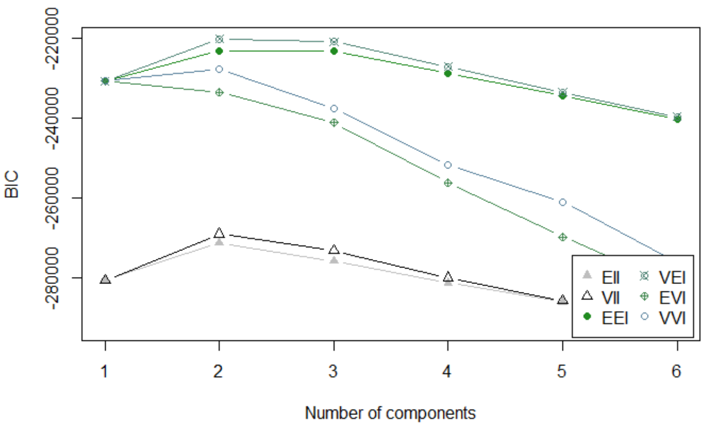

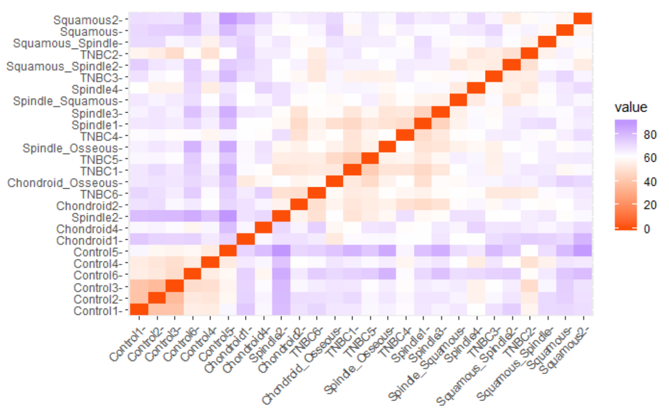

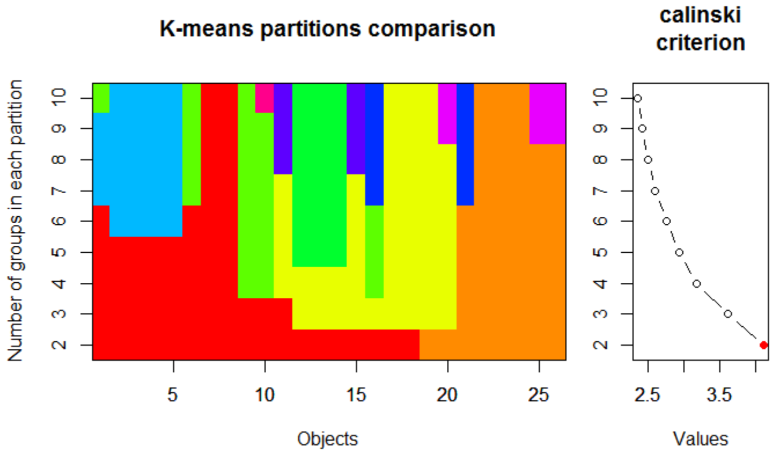

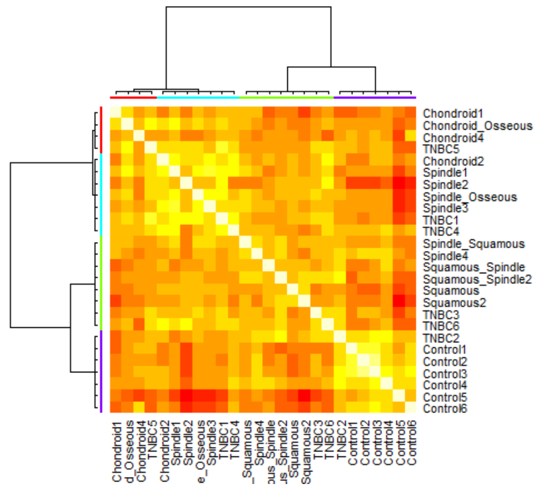

k=2

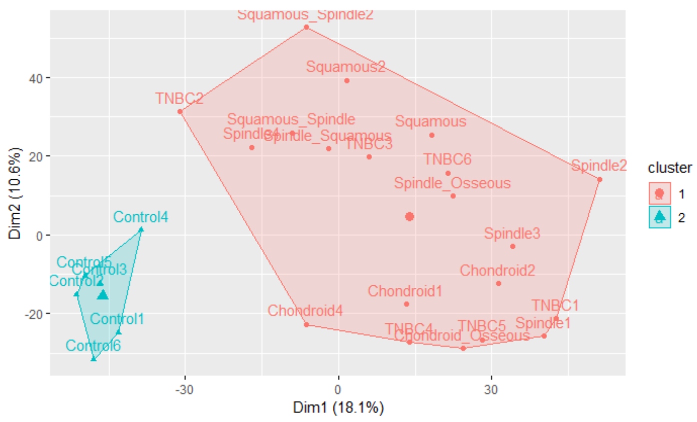

k=3

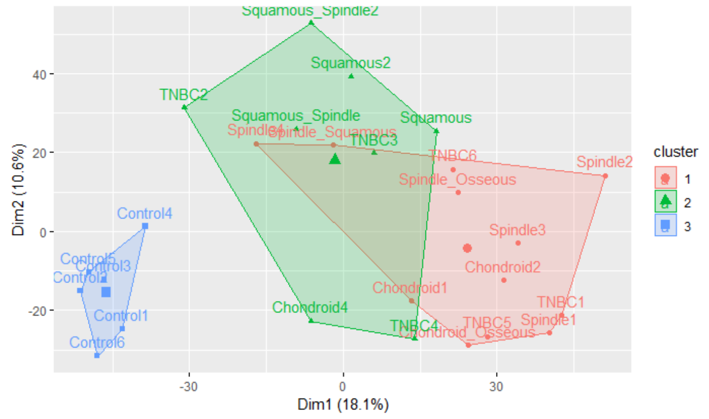

k=4

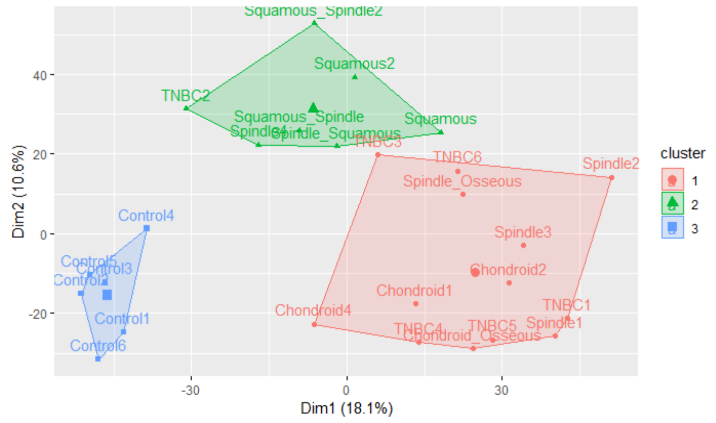

k=5

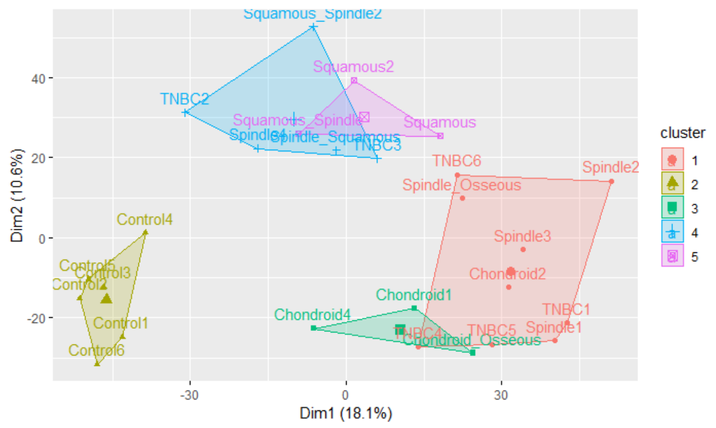

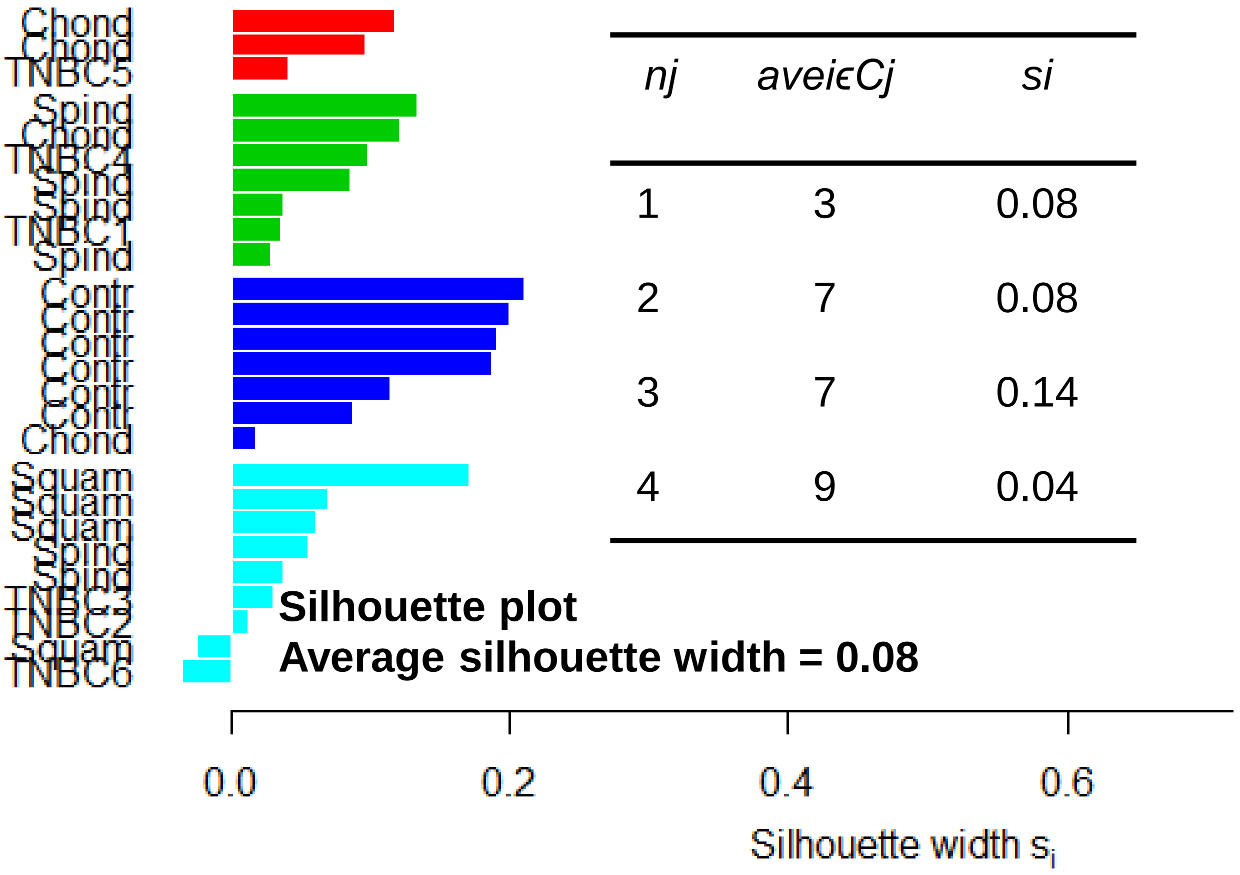

nj	aveiєCj	si
1	3	0.08
2	7	0.08
3	7	0.14
4	9	0.04

k=2	k=3	k=4	k=5
Normal<br>Tumor	Normal<br>MBC+TN<br>MBC+TN	Normal<br>Spindle + TN<br>Squamous + TN<br>Chondroid	Normal<br>Spindle + TN<br>Squamous<br>Chondroid<br>Mixed

Supplementary Figure 2. Summary of unsupervised k-means methods for determining optimal clusters. Table shows summary of results from seven different algorithms used in R (Bayesian, within-cluster sum of squares (WSS), Vegan, Elbow, Gap statistic, Silhouette, and Apcluster) for determining the optimal number of clusters among our cohort of patient tumor samples. Representative plots (top right) show hierarchical clustering heatmaps and a range of 2-4 optimal clusters found. Representative silhouette plot with 4 clusters shows an average silhouette width of 0.08, where silhouette ranges from -1 to 1. PCA plots (left) and table of corresponding histological subtypes (bottom right) demonstrate a distinction between normal and tumor (MBC and TNBC) with 2-3 clusters present, and a distinction between both normal and MBC subtypes (spindle, squamous, sarcomatoid) with 4-5 clusters, where triple-negative (TNBC) clustered with spindle or squamous MBC.

Supplementary Figure 3

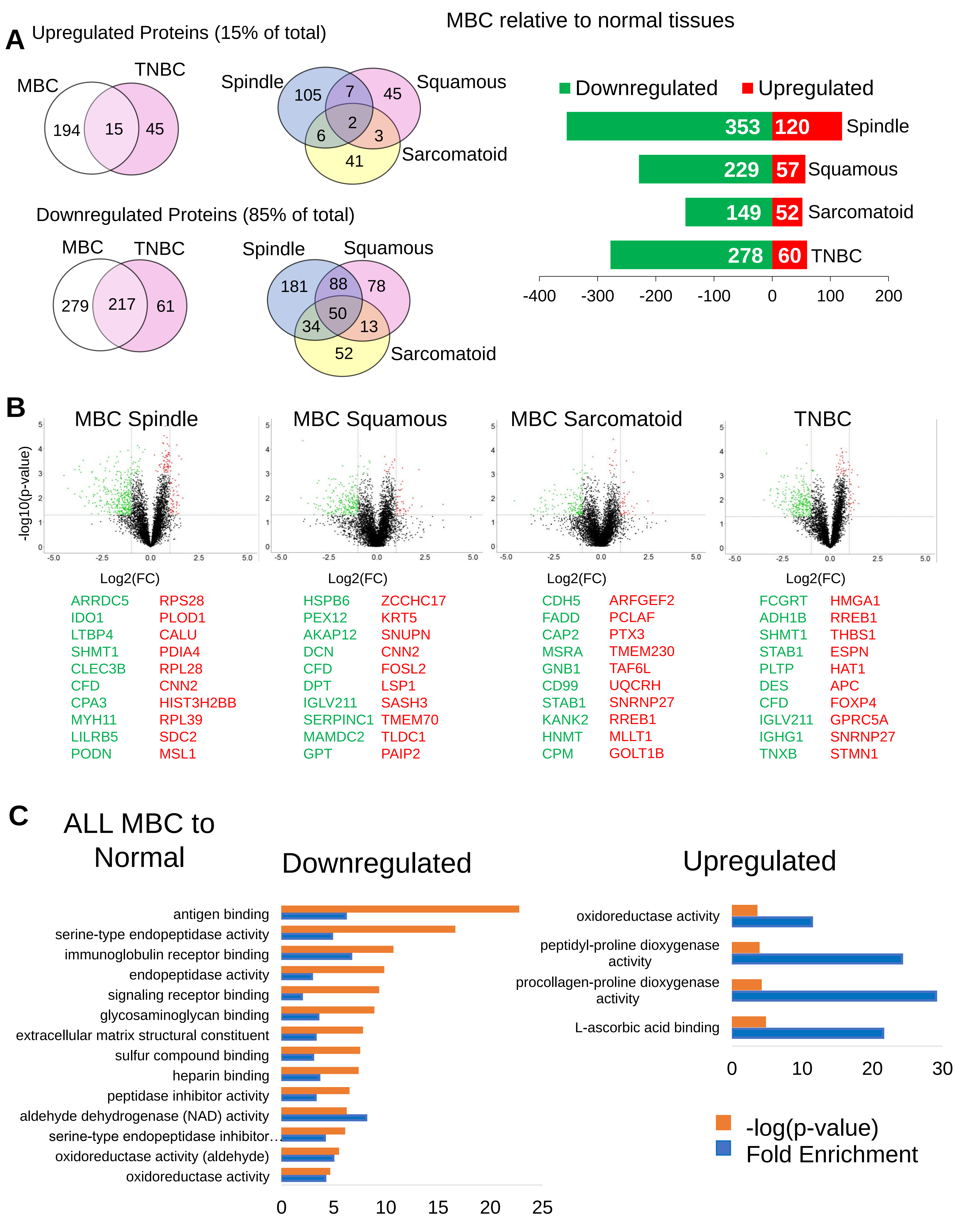

Supplementary Figure 3. Supervised differential expression analysis reveals unique MBC protein signatures by histological subtype relative to normal breast.

A. Total differentially expressed proteins in MBC and TNBC relative to normal tissues. Patient samples were grouped according to their predominant histological MBC subtype (spindle, squamous, and sarcomatoid), and considered proteins of $p<0.05$ and $FC>1$, and highly significant outliers of $p<0.001$.

B. Statistical analysis shows volcano plots for MBC spindle, squamous and sarcomatoid and triple-negative (TNBC) subtypes as log2-Fold change versus –log10(p-value), where proteins indicated in green are downregulated and red indicates upregulated proteins. Horizontal boundary represents $p=0.05$ and two vertical boundaries indicate FC = 1 and -1. The list of the top 10 most significant up- and downregulated proteins are shown.

C. Enrichment analyses of all MBC relative to normal breast tissues demonstrating up- and downregulated enriched terms from gene ontology (GO) using PANTHER (v14.1) database. Fold enrichment scores show the GO annotation for biological process and the –log(p-value).

Supplementary Figure 4

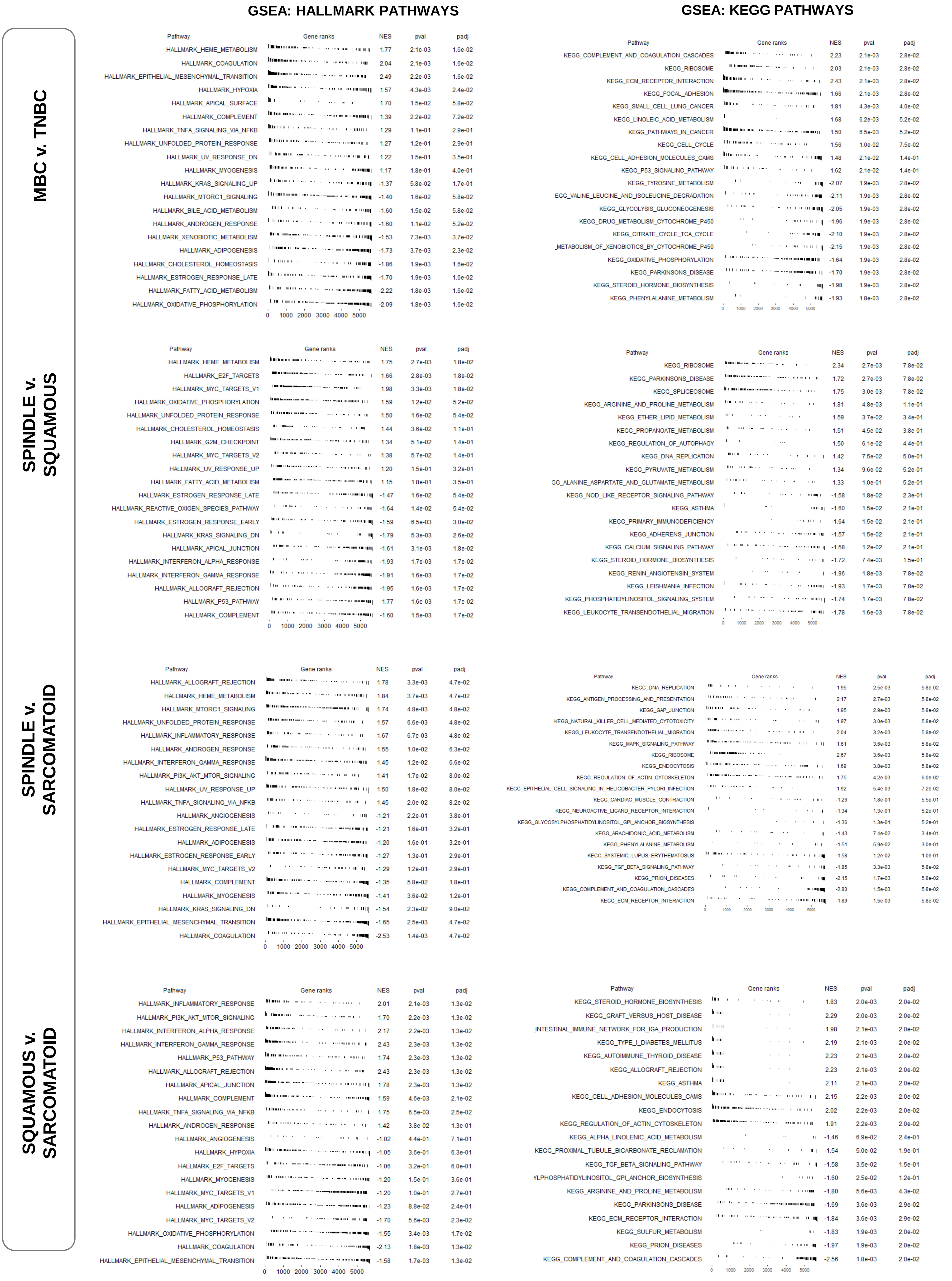

Supplementary Figure 4. Top pathways of gene set enrichment analysis (GSEA) in Hallmark and KEGG gene sets within MBC and relative to TNBC. Ranked lists of gene sets were previously attained from differential expression analysis for each disease condition (MBC vs. TNBC, and across MBC subgroups (Spindle vs Squamous, Spindle vs Sarcomatoid, and Squamous vs. Sarcomatoid) to run the GSEA analysis using MSigDB against hallmark gene sets and KEGG (Kyoto Encyclopedia of Genes and Genomes). Tables show top pathways, gene ranks, normalized enrichment score (NES), pvalue, and padjust value, where $p<0.05$ was considered significant.

Supplementary Figure 5

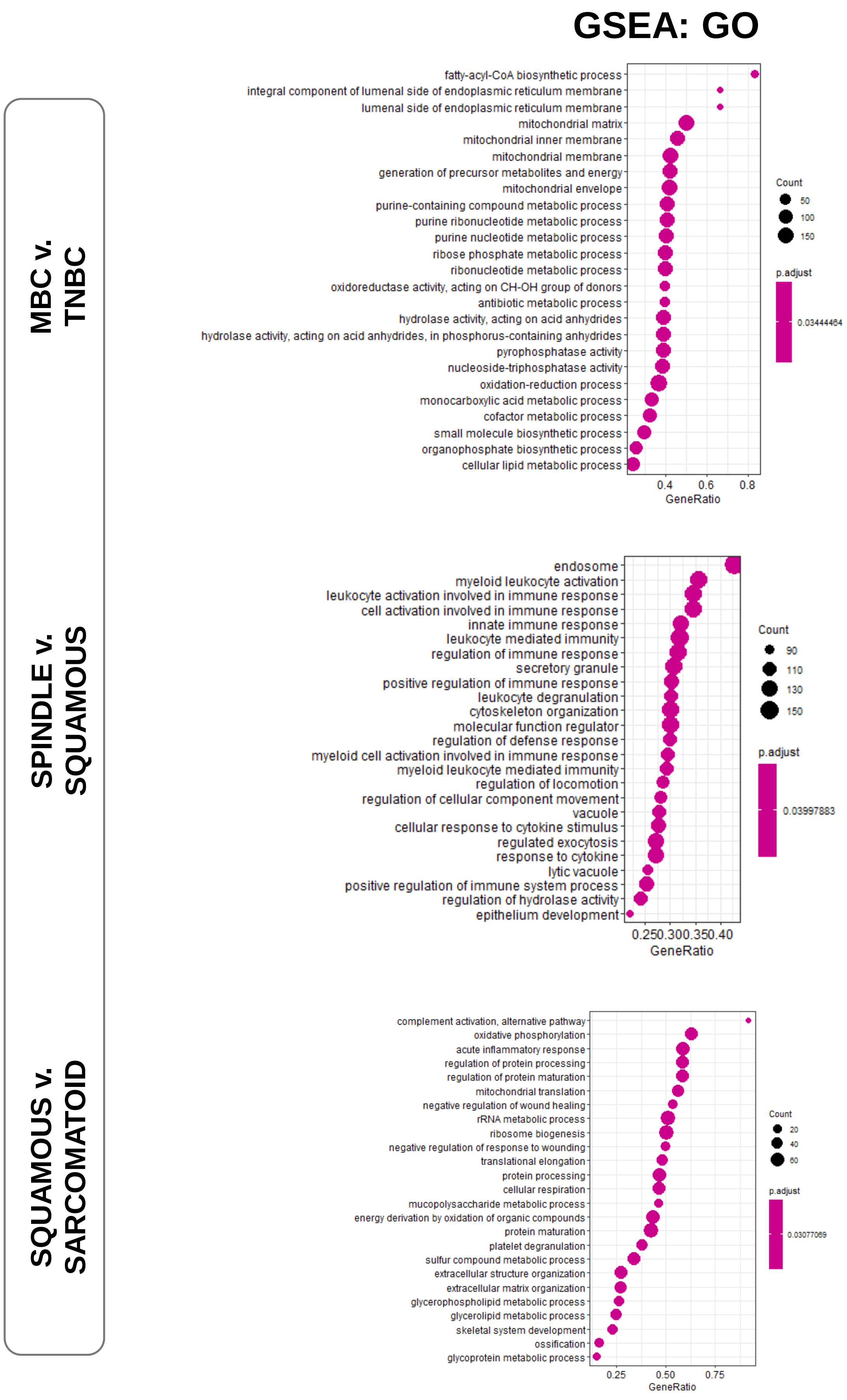

* Note: No statistically enriched terms found between Spindle vs. Sarcomatoid

Supplementary Figure 5. GSEA analysis of curated GO gene sets (C5) from MSigDB for MBC relative to TNBC, spindle vs squamous, and squamous vs. sarcomatoid. Bar plot shows the total number of differentially expressed up- and downregulated proteins in each category. Networks display most significant enriched terms from the GSEA: GO analysis including biological process, molecular function, or cellular component for each carcinoma condition. Node size depicts protein count (i.e. gene count), and all terms are significant and filtered to padjust<0.05. No significant terms were found between spindle and sarcomatoid MBC.

Supplementary Figure 6

ENRICHMENT NETWORK: GSEA: Hallmark GO Over-representation

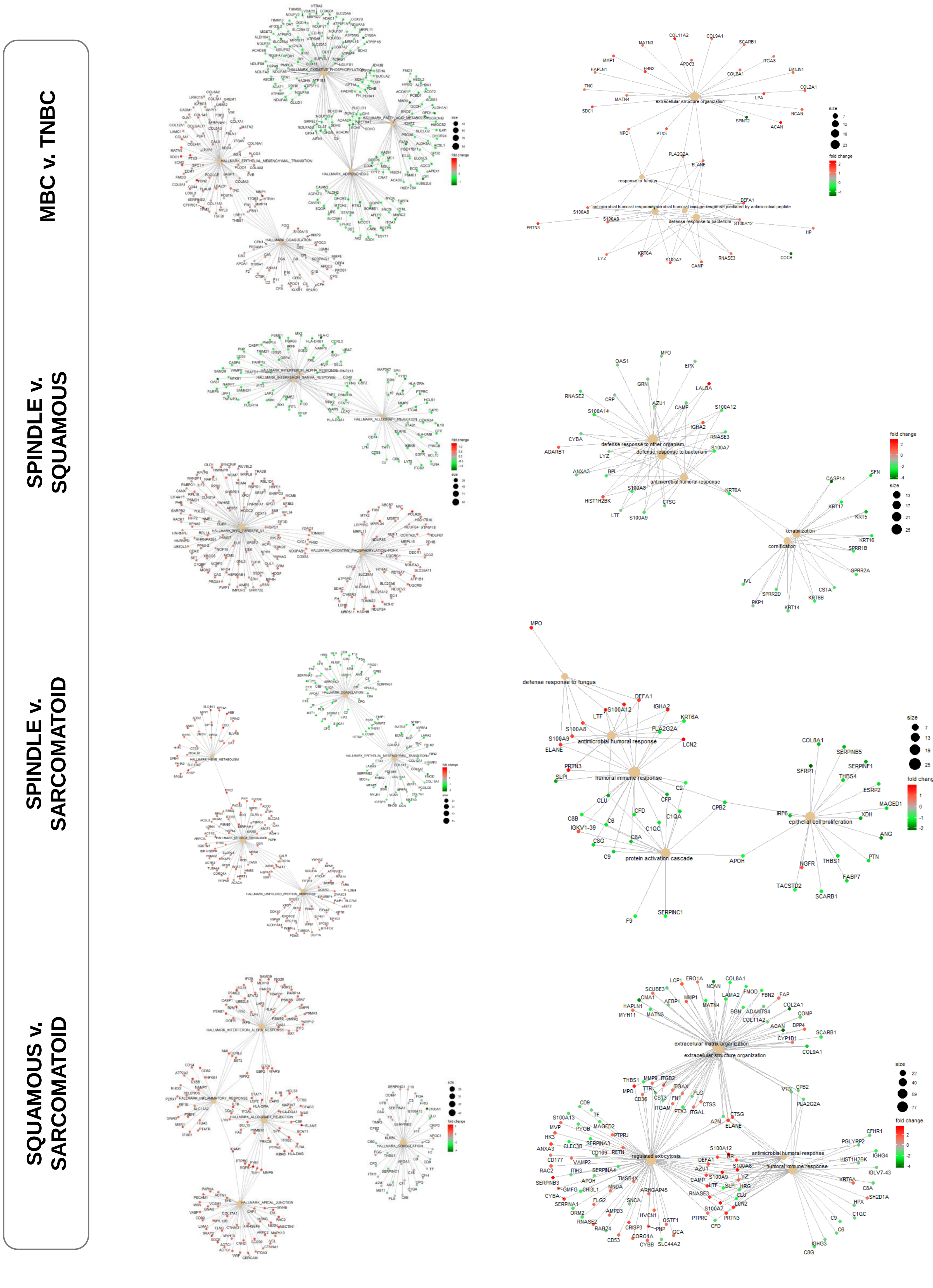

Supplementary Figure 6. Enriched protein networks of the top GSEA hallmarks and GO terms within MBC and relative to TNBC. Networks display most significant enriched terms and associated proteins for each carcinoma condition. Node size depicts protein count, and the color scheme are fold change values (log2FC), where red=upregulated and green=downregulated proteins. All terms were filtered at pval<0.05 and padj<0.05.

Supplementary Figure 7

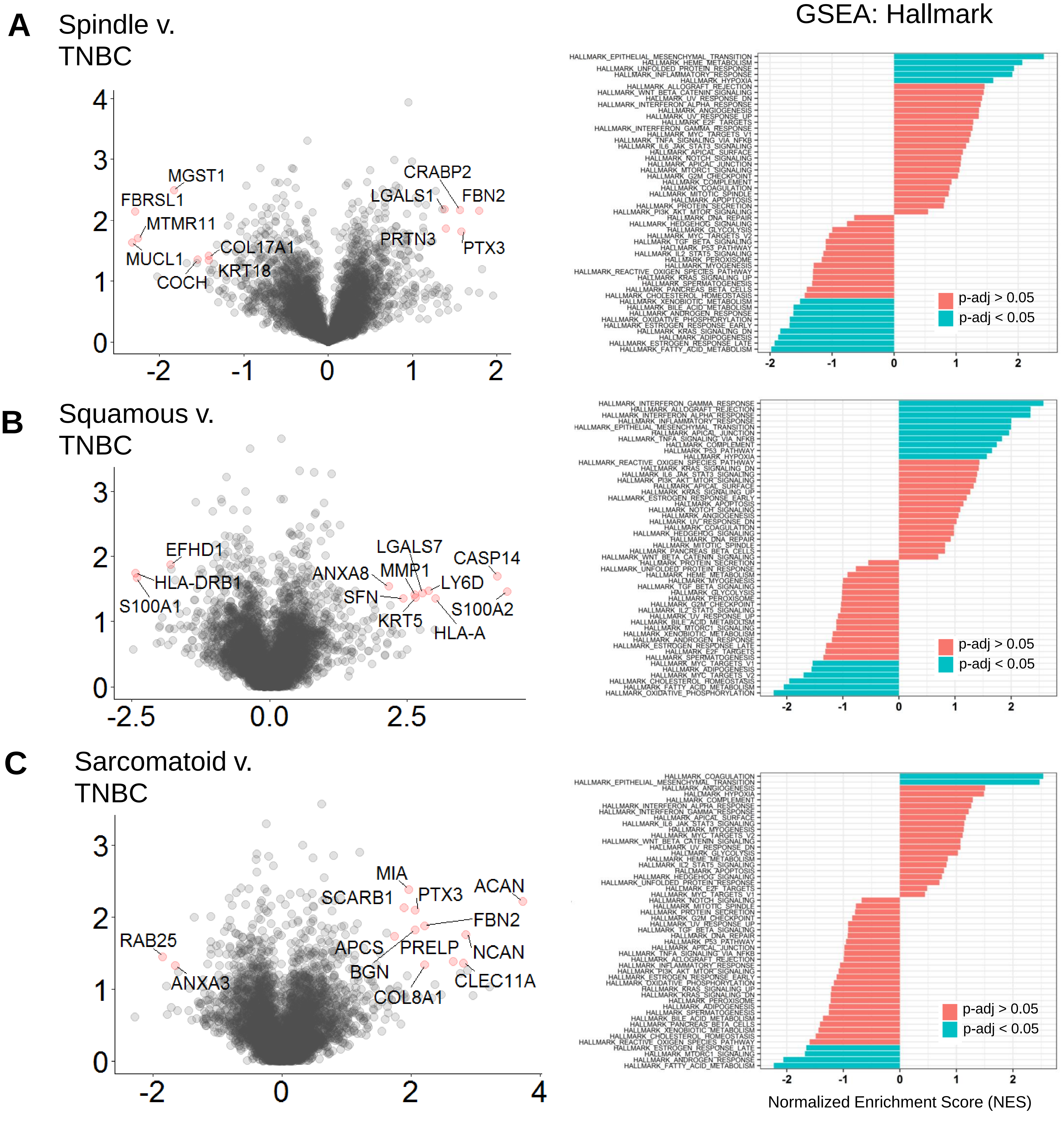

Supplementary Figure 7. Differential expression analysis between TNBC and all MBC subtypes. Volcano plots for spindle MBC vs. TNBC, squamous MBC vs. TNBC, and sarcomatoid MBC vs TNBC, as log2-Fold change versus –log10(p-value) and GSEA hallmark analysis (MSigDB v7.0), where top pathways are shown as p-adj <0.05 (blue). We followed the clusterProfiler package in R with n=1000 permutations and FDR <0.05.

Supplementary Figure 8

A

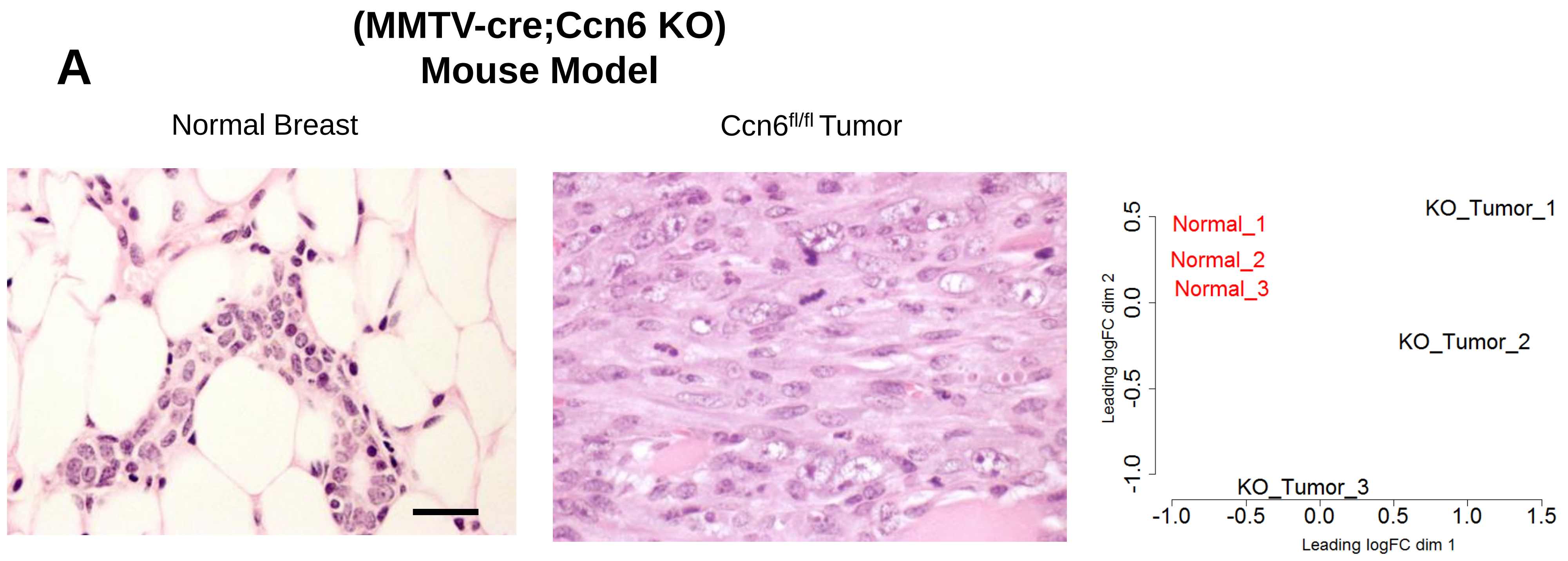

B

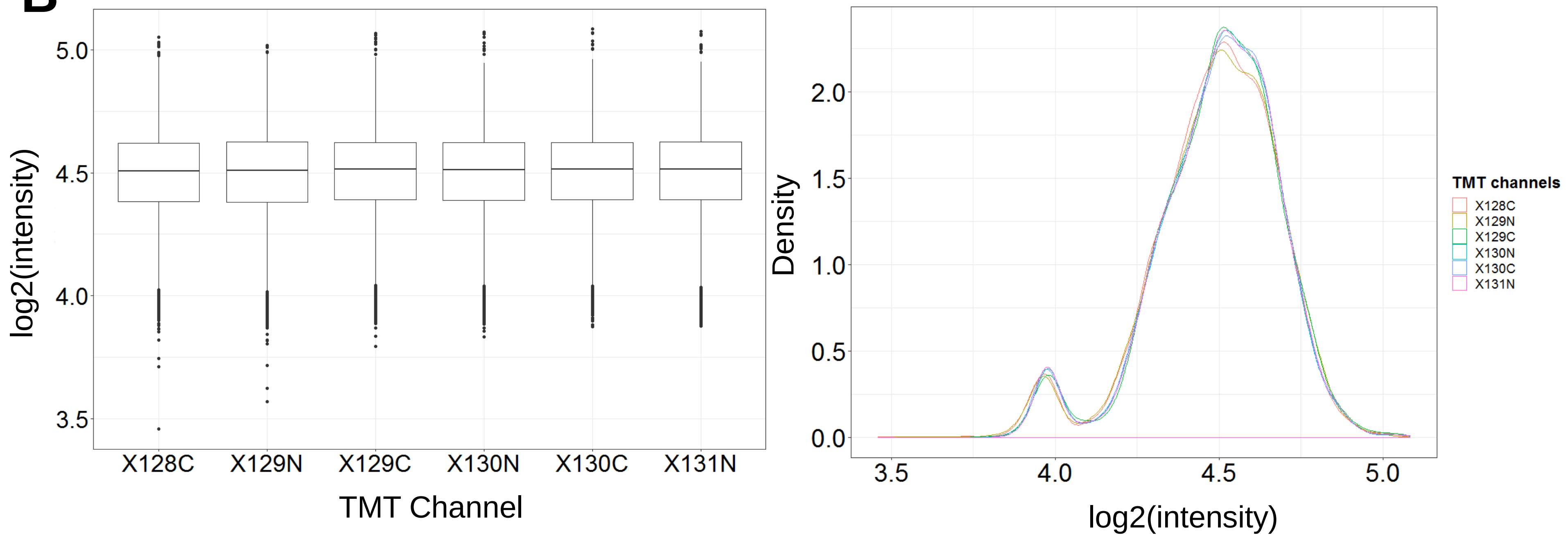

C

Pathway	Gene ranks	NES	pval	padj
HALLMARK_UNFOLDED_PROTEIN_RESPONSE		1.82	3.4e-03	2.4e-02
HALLMARK_MYOGENESIS		1.70	4.7e-03	2.9e-02
HALLMARK_INFLAMMATORY_RESPONSE		1.70	5.8e-03	3.1e-02
HALLMARK_MYC_TARGETS_V1		2.15	6.2e-03	3.1e-02
HALLMARK_E2F_TARGETS		1.69	8.4e-03	3.8e-02
HALLMARK_MYC_TARGETS_V2		1.70	1.1e-02	4.4e-02
HALLMARK_EPITHELIAL_MESENCHYMAL_TRANSITION		1.38	2.2e-02	7.7e-02
HALLMARK_P53_PATHWAY		1.40	3.1e-02	1.0e-01
HALLMARK_TNFA_SIGNALING_VIA_NFKB		1.37	5.6e-02	1.4e-01
HALLMARK_IL6_JAK_STAT3_SIGNALING		1.46	6.1e-02	1.5e-01
HALLMARK_INTERFERON_ALPHA_RESPONSE		-1.45	4.9e-02	1.4e-01
HALLMARK_CHOLESTEROL_HOMEOSTASIS		-1.47	4.8e-02	1.4e-01
HALLMARK_HEME_METABOLISM		-1.37	4.7e-02	1.4e-01
HALLMARK_ESTROGEN_RESPONSE_LATE		-1.54	1.1e-02	4.4e-02
HALLMARK_REACTIVE_OXIGEN_SPECIES_PATHWAY		-1.87	3.1e-03	2.4e-02
HALLMARK_BILE_ACID_METABOLISM		-2.08	1.5e-03	1.5e-02
HALLMARK_PEROXISOME		-1.96	1.4e-03	1.5e-02
HALLMARK_XENOBIOTIC_METABOLISM		-2.06	1.3e-03	1.5e-02
HALLMARK_FATTY_ACID_METABOLISM		-2.37	1.3e-03	1.5e-02
HALLMARK_ADIPOGENESIS		-2.54	1.2e-03	1.5e-02

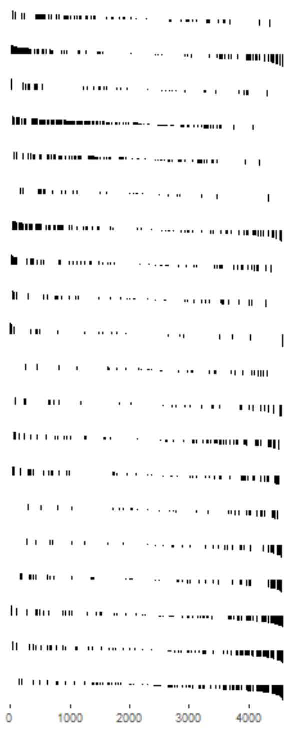

Supplementary Figure 8. Pre-processing of LC-MS/MS TMT 10-plex proteomics of MBC mouse model (MMTV-cre;Ccn6 KO).
A. Representative histology images stained with hematoxylin and eosin (H&E) of normal mouse breast tissues and MMTV-cre;Ccn6 mouse tumors. Scale bar = 50 μm. Raw data distribution of 6 mouse samples, n=3 normal breast tissues (red) and n=3 CCN6 knockout tumors (black).
B. TMT channel vs log2(intensity) for all samples, Density distribution plot of log2-intensity vs. density of all expression values per sample.
C. top hallmark pathways from GSEA analysis using MSigDB. Table shows gene ranks, normalized enrichment score (NES), pvalue, and padjust value, where $p<0.05$ was considered significant.

Supplementary Table 1. LC-MS/MS study design and tandem mass tag (TMT) designations for patient samples

SAMPLE ID	HISTOLOGICAL SUBTYPE	SET	TMT CHANNEL
MBC (C1)	Sarcomatoid (Chondroid)	A	126
MBC (C2)	Sarcomatoid (Chondroid)	A	127N
MBC (Sp1)	Spindle	A	127C
MBC (Sq1)	Squamous	A	128N
MBC (Sq2)	Squamous	A	128C
TNBC1	Triple-negative	A	129N
TNBC2	Triple-negative	A	129C
Control1	Normal breast	A	130N
Control2	Normal breast	A	130C
Master Mix	Reference Sample	A	131
MBC (C3)	Sarcomatoid (Chondroid)	B	126
MBC (C4)	Sarcomatoid	B	127N
MBC (Sp2)	(Chondroid/Osseous)	B	127C
MBC (Sp3)	Spindle	B	128N
MBC (Sq3)	Spindle/Squamous	B	128C
TNBC3	Squamous	B	129N
TNBC4	Triple-negative	B	129C
Control3	Triple-negative	B	130N
Control4	Normal breast	B	130C
Master Mix	Normal breast	B	131
MBC (C5)	Reference Sample	C	126
MBC (Sp4)	Sarcomatoid (Chondroid)	C	127N
MBC (Sp5)	Spindle/Osseous	C	127C
MBC (Sq4)	Spindle	C	128N
MBC (Sp6)	Squamous	C	128C
TNBC5	Spindle	C	129N
TNBC6	Triple-negative	C	129C
Control5	Triple-negative	C	130N
Control6	Normal breast	C	130C
Master Mix	Normal breast	C	131
	Reference Sample		

Supplementary Table 1. LC-MS/MS study design and tandem mass tag (TMT) designations for patient samples. Table shows a summary of all samples used in the LC-MS/MS proteomics study design, with tandem mass tag (TMT) designations for patient samples, experiments 1, 2 and 3 given as Set A, B, and C, respectively.

Supplementary Table 2. Summary of key enriched features in MBC spindle, squamous and sarcomatoid versus non-MBC triple-negative tumors relative to normal tissues.

Carcinoma	Enriched Altered Process	Cellular Component	Major Domain
**Spindle v. Normal**<br>UP	Translation (1.03E-45)<br>Metabolic process (3.7E-22)<br>RNA processing (2.97E-08)<br>Peptidyl-proline modification (4.03E-05)<br>DNA conformation change (0.003)	Ribosome (2.03E-36)<br>Cytoplasm (3.35E-16)<br>ER (1.22E-09)<br>Nucleus (9.19E-06)	Prolyl 4-Hydroxylase<br>Thioredoxin<br>Oxoglutarate<br>20G-Fe(II) oxygenase<br>Threonyl/Alanyl tRNA synthetase
DOWN	Acute inflammatory response (2.97E-21)<br>Proteolysis (6.37E-16)<br>Immune response (5.84E-15)<br>Extracellular structure organization (2.3E-13)<br>Coagulation (4.21E-11)<br>Oxidoreductase metabolic activity (3.53E-10)<br>Wound healing (5.3E-10)<br>Peptidase activity (1.81E-10)	Extracellular region (3.29E-41)<br>Cytoplasmic vesicle (2.48E-14)<br>Extracellular matrix (2.82E-11)<br>ER (5.81E-13)<br>Protein-lipid complex (2.39E-09)<br>High-density lipoprotein (1.75E-07)<br>Caveola (0.00015)	Serpin family<br>vWF type A<br>Peptidase S1<br>Aldehyde dehydrogenase<br>Thrombospondin<br>EGF-like<br>Immunoglobulin<br>Lipocalin<br>PTRF/SDPR family<br>Caveolin
**Squamous v. Normal**<br>UP	Neutrophil degranulation (3.59E-10)<br>Leukocyte activation (5.29E-08)<br>Immune response (2.32E-06)<br>Cell migration (epithelial, endothelial, leukocyte) (0.0001)<br>Keratinocyte differentiation (0.0025)<br>Inflammatory response (0.0027)<br>Cytoskeleton organization (0.0057)	Secretory vesicle (5.19E-09)<br>Cytoplasm (7.08E-06)<br>Cytoskeleton (0.00084)<br>Intermediate filament (0.0032)	S-100/ICaBP Calcium binding<br>Intermediate filament protein
DOWN	Oxoacid metabolic process (3.68E-09)<br>Extracellular structure organization (9.76E-06)<br>Vasculature development (4.6E-05)<br>Peptidase activity (7.62E-05)<br>Proteolysis (0.00016)<br>Cell adhesion (0.0014)	Extracellular region (5.18E-20)<br>Cytoplasm (1.85E-09)<br>Extracellular matrix (1.63E-07)<br>Secretory granule (8.32E-05)<br>Cell surface (0.00076)<br>Plasma lipoprotein (0.0035)	Aldehyde dehydrogenase<br>Lipocalin /fatty-acid<br>Cavin family<br>Cystatin<br>EGF-like<br>Serpin family<br>Thrombospondin
**Sarcomatoid v. Normal**<br>UP	Extracellular structure organization (1.68E-06)<br>Extracellular matrix organization (2.76E-06)<br>Hyaluronic acid binding (0.00036)<br>Phosphatidylserine binding (0.00091)<br>Collagen V binding (0.0038)<br>Glycosaminoglycan binding (0.0038)	Extracellular region (0.00016)<br>Intracellular organelle (0.00013)<br>ER lumen (0.00046)	vWF type C<br>EGF<br>Extracellular link<br>Thrombospondin<br>Collagen<br>C type lectin<br>Histone H2B
DOWN	Oxidation-reduction process (1.1E-09)<br>Oxoacid metabolic process (1.13E-06)<br>Alcohol metabolic process (1.7E-06)<br>Exocytosis (0.00031)<br>Angiotensin maturation (0.009)<br>Actin filament capping (0.0097)	Cytoplasm (1.09E-09)<br>Secretory granule (3.71E-06)<br>Lipid droplet (3.71E-06)<br>Cytosol (0.00056	Aldehyde dehydrogenase<br>Cavin family<br>NAD(P)-binding<br>Zinc carboxypeptidase<br>vWF<br>Immunoglobulin
**TNBC v. Normal**<br>UP	mRNA processing (2.37E-05)<br>mRNA metabolic process (8.66E-05)<br>RNA splicing/processing (0.00019)<br>DNA conformation change (0.0028)<br>Chromatin assembly (0.0135)<br>Microtubule cytoskeleton organization (0.0166)<br>Cell cycle process (0.0235)<br>Spindle organization (0.0257)	Intracellular organelle (6.03E-07)<br>Nuclear part (6.03E-07)<br>Nucleus (4.08E-06)<br>Fibrillar center (0.001)<br>Ribonucleoprotein complex (0.0066)<br>Cytoskeletal part (0.0076)<br>Filamentous actin (0.013)	Thrombospondin<br>RNA recognition motif<br>RanBP1<br>RNA-binding superfamily<br>TSP type-3 repeat<br>Laminin G<br>Nucleotide-binding
DOWN	Extracellular structure organization (1.82E-13)<br>Exocytosis (1.56E-10)<br>Immune / inflammatory response (6.32E-08)<br>Cell adhesion, proteolysis (4.02E-07)<br>Wound healing (3.14E-05)<br>Primary metabolic process (1.29E-06)	Extracellular region (2.67E-33)<br>Secretory granule (8.92E-15)<br>ER (1.78E-10)<br>Cell surface (4.95E-07)<br>Cytoplasm (8.28E-07)<br>High-density lipoprotein (2.74E-06)	Serpin family<br>vWF<br>Collagen<br>Aldehyde dehydrogenase<br>Immunoglobulin<br>Thrombospondin<br>Caveolin
