# Supplementary material for: Quantitative proteomic landscape of metaplastic breast carcinoma pathological subtypes and their relationship to triple-negative tumors
Source: Nat Commun. 2020 Apr 7;11:1723. doi: 10.1038/s41467-020-15283-z (PMC7138853; doi:10.1038/s41467-020-15283-z)
Supplement: Supplementary file 3 — Description of Additional Supplementary Information [file 41467_2020_15283_MOESM3_ESM.docx]

**Description of Additional Supplementary Files**

File name: Supplementary Data 1

Description: GSEA analysis of MBC vs TNBC using the MSigDB for curated gene sets of pathway databases (C2) and GO gene sets (C5).

File name: Supplementary Data 2

Description: GSEA analysis of Spindle vs. Squamous using the MSigDB for curated gene sets of pathway databases (C2) and GO gene sets (C5).

File name: Supplementary Data 3

Description: GSEA analysis of Spindle vs. Sarcomatoid using the MSigDB for curated gene sets of pathway databases (C2) and GO gene sets (C5).

File name: Supplementary Data 4

Description: GSEA analysis of Squamous vs. Sarcomatoid using the MSigDB for curated gene sets of pathway databases (C2) and GO gene sets (C5).
