# Supplementary material for: Quantitative proteomic landscape of metaplastic breast carcinoma pathological subtypes and their relationship to triple-negative tumors
Source: Nat Commun. 2020 Apr 7;11:1723. doi: 10.1038/s41467-020-15283-z (PMC7138853; doi:10.1038/s41467-020-15283-z)
Supplement: Supplementary file 8 — Reporting Summary [file 41467_2020_15283_MOESM8_ESM.pdf]

nature research

Corresponding author(s): Alexey I. Nesvizhskii, Celina G. Kleer

Last updated by author(s): Feb 27, 2020

Reporting Summary

Nature Research wishes to improve the reproducibility of the work that we publish. This form provides structure for consistency and transparency in reporting. For further information on Nature Research policies, see Authors & Referees and the Editorial Policy Checklist.

Statistics

For all statistical analyses, confirm that the following items are present in the figure legend, table legend, main text, or Methods section.

n/a	Confirmed	
☐	☒	The exact sample size (*n*) for each experimental group/condition, given as a discrete number and unit of measurement
☐	☒	A statement on whether measurements were taken from distinct samples or whether the same sample was measured repeatedly
☐	☒	The statistical test(s) used AND whether they are one- or two-sided<br>*Only common tests should be described solely by name; describe more complex techniques in the Methods section.*
☐	☒	A description of all covariates tested
☐	☒	A description of any assumptions or corrections, such as tests of normality and adjustment for multiple comparisons
☐	☒	A full description of the statistical parameters including central tendency (e.g. means) or other basic estimates (e.g. regression coefficient) AND variation (e.g. standard deviation) or associated estimates of uncertainty (e.g. confidence intervals)
☐	☒	For null hypothesis testing, the test statistic (e.g. *F*, *t*, *r*) with confidence intervals, effect sizes, degrees of freedom and *P* value noted<br>*Give P values as exact values whenever suitable.*
☐	☒	For Bayesian analysis, information on the choice of priors and Markov chain Monte Carlo settings
☐	☒	For hierarchical and complex designs, identification of the appropriate level for tests and full reporting of outcomes
☒	☐	Estimates of effect sizes (e.g. Cohen's *d*, Pearson's *r*), indicating how they were calculated

Our web collection on statistics for biologists contains articles on many of the points above.

Software and code

Policy information about availability of computer code

Data collection	Xcalibur (v4.1.31.19) for data collection and MSConvert in Proteowizard software suite (v3.0.18208-7b41a1f82) to process raw MS data. For WES data acquisition, DNA samples from 10 pairs of tumor and healthy samples from this patient cohort were selected, and the NovaSeq 6000 Illumina system (150 bp paired-end format), libraries prepared with NEBNext Ultra 2 FS DNA library prep kit for Illumina. The IDT xGen hybridization capture kit was used and samples processed with Illumina's bcl2fastq software (v2.20).
Data analysis	Philosopher (github.com/Nesvilab/philosopher) and TMT-Integrator (github.com/Nesvilab/tmt-integrator) for proteomics analysis. FastQC (v0.11.7), Trimmomatic (v0.39), BWA (v0.7.17), GATK (v4.1.4.0 or v3.8), MuTect2, Varscan (v2.4.4), and Strelka (v2.9.10) were used for WES analysis. For enrichment analysis, we used either GO over-representation tests in PANTHER (v14.1) and STRING (v11.0), GSEA from BROAD Institute according to GSEA documentation using MSigDB (v7.0), and WEB-based GEne SeT AnaLysis Toolkit (2019).

For manuscripts utilizing custom algorithms or software that are central to the research but not yet described in published literature, software must be made available to editors/reviewers. We strongly encourage code deposition in a community repository (e.g. GitHub). See the Nature Research guidelines for submitting code & software for further information.

Data

Policy information about availability of data

All manuscripts must include a data availability statement. This statement should provide the following information, where applicable:

- Accession codes, unique identifiers, or web links for publicly available datasets
- A list of figures that have associated raw data
- A description of any restrictions on data availability

Proteomics data are available via ProteomeXchange with identifier PXD014414. For WES data, at this time, informed consent from MBC patients does not allow for public deposition but can be communicated upon reasonable request to C.K.

Field-specific reporting

Please select the one below that is the best fit for your research. If you are not sure, read the appropriate sections before making your selection.

☒ Life sciences ☐ Behavioural & social sciences ☐ Ecological, evolutionary & environmental sciences

For a reference copy of the document with all sections, see nature.com/documents/nr-reporting-summary-flat.pdf

Life sciences study design

All studies must disclose on these points even when the disclosure is negative.

Sample size	From an original batch of 28 frozen tissue samples from women with MBC, we selected 15 samples, where 1 was later excluded upon analysis. We assembled a clinical cohort of 14 frozen MBCs which were classified clinically according to their predominant metaplastic component into the following subtypes: spindle (n=6), squamous (n=4), and sarcomatoid (n=4). We also included 6 non-metaplastic TNBCs and 6 normal breast tissues. The rationale for appropriate sample sizes for biological tissues considered the following criteria: 1) Requirement to have at least n=3 samples or more, and 2) Equal numbers of samples from each tumor type be consistent across each of the three TMT experiments, which was limited to 10 samples per experiment.
Data exclusions	We excluded 1 sample from the available proteomics data set (see Table 1, Case #5; Histological subtype: chondroid). The pathologist sectioned a piece of this sample and was later confirmed to be "normal tissue" in both histology and proteomics, not a tumor piece as intended, and consequently excluded from data analyses.
Replication	The data are available via PRIDE ProteomeXchange for verifying reproducibility of results. All patients were women with a mean age of 55 years old (range 33 to 89 years old). The majority (14 of 15, 93.33%) MBC and all TNBC were of histological grade 3, and all were negative for estrogen and progesterone receptor, and for HER2/neu overexpression. Of the 14 MBC, 11 (78.6%) were stage I/II and 3 (21.4%) stage III/IV at the time of diagnosis. Of the 6 TNBC, 5 (83.3%) were stage I/II and 1 (16.6%) stage III at diagnosis. At follow up, 4 of 14 (28.6%) MBC, and 1 of 6 (16.66%) TNBC developed distant metastasis to the lungs, liver, skin, and bone. The current study did not use technical replicates of biological samples, however all necessary measures were taken to verify reproducibility of results. Through cross-examination of differential expression analysis and GSEA tests from multiple databases, and comparisons between all possible tumor types relative to each other and all tumor types relative to normal tissues we were successful in replicating the top pathways and/or proteins involved in their unique signatures discovered per tumor subtype.
Randomization	We arranged the samples into three experimental groups appropriate for the TMT10plex isobaric labeling strategy (n=10 samples per group; 9 individual tissue samples and one common sample consisting of a pool of all 26 tissues, as a reference sample). Among the 9 tissue samples that could be employed per TMT experiment, 5 were MBC tumors, and the remaining 4 were covariates consisting of n=2 non-MBC (TNBC) tumors and n=2 non-tumor, normal breast tissues.
Blinding	Participants of this study were not blinded, however initial tissue collection was randomized or blinded. For data acquisition and data analysis, blinding was not necessary or advised to reduce bias of statistical analyses. Investigators were aware of assigned sample designations to enable statistical power across experiments was met. Metaplastic carcinomas were collected from the surgical pathology files at Johns Hopkins University and control samples were collected from the surgical pathology files at the University of Michigan, with IRB approval.

Reporting for specific materials, systems and methods

We require information from authors about some types of materials, experimental systems and methods used in many studies. Here, indicate whether each material, system or method listed is relevant to your study. If you are not sure if a list item applies to your research, read the appropriate section before selecting a response.

Materials & experimental systems

n/a	Involved in the study	
☒	☐	Antibodies
☒	☐	Eukaryotic cell lines
☒	☐	Palaeontology
☐	☒	Animals and other organisms
☒	☐	Human research participants
☐	☒	Clinical data

Methods

n/a	Involved in the study	
☒	☐	ChIP-seq
☒	☐	Flow cytometry
☒	☐	MRI-based neuroimaging

Animals and other organisms

Policy information about studies involving animals; ARRIVE guidelines recommended for reporting animal research

Laboratory animals	Adult virgin female FVB mice (4-8 months of age) were employed.
Wild animals	not applicable

Field-collected samples	not applicable
Ethics oversight	The mouse work was approved by the University of Michigan UCUCA under protocol PRO00009007.

Note that full information on the approval of the study protocol must also be provided in the manuscript.

Clinical data

Policy information about clinical studies

All manuscripts should comply with the ICMJE guidelines for publication of clinical research and a completed CONSORT checklist must be included with all submissions.

Clinical trial registration	No clinical trials were conducted. We performed a retrospective analysis of tissue samples that would otherwise be discarded.
Study protocol	Oversight for the study protocol was determined by the corresponding authors.
Data collection	All patient samples were collected by Surgical Pathology at Johns Hopkins Hospital between April 1996 and June 2015. These tissues were de-identified and would have otherwise be discarded.
Outcomes	Outcomes from an initial randomized batch of 28 frozen tissue samples of MBC tumors, we assembled a clinical cohort of 15 human MBC samples.
